# Development of Pyramidal Microwells for Enhanced Cell Spheroid Formation in a Cell-on-Chip Microfluidic System for Cardiac Differentiation of Mouse Embryonic Stem Cells

**DOI:** 10.3390/cells13242132

**Published:** 2024-12-23

**Authors:** Tepparit Wongpakham, Thanapat Chunfong, Wutthinan Jeamsaksiri, Kriengkai Chessadangkul, Sudchaya Bhanpattanakul, Wirakan Kallayanathum, Theerawat Tharasanit, Alongkorn Pimpin

**Affiliations:** 1 Department of Mechanical Engineering, Faculty of Engineering, Chulalongkorn University, Bangkok 10330, Thailand; tepparit_book1@yahoo.com (T.W.); chunfong_t@hotmail.com (T.C.); xunday1024@gmail.com (K.C.); 2 Thai Microelectronics Center, National Electronics and Computer Technology Center, Chachoengsao 24000, Thailand; wutthinan.jeamsaksiri@nectec.or.th; 3 Department of Pathology, Faculty of Veterinary Science, Chulalongkorn University, Bangkok 10330, Thailand; sudchaya73@gmail.com; 4 Center of Excellence for Veterinary Clinical Stem Cells and Bioengineering, Chulalongkorn University, Bangkok 10330, Thailand; 5 Department of Obstetrics, Gynaecology and Reproduction, Faculty of Veterinary Science, Chulalongkorn University, Bangkok 10330, Thailand; wirakan.mind@gmail.com; 6 Micro/Nano Electromechanical Integrated Device Research Unit, Faculty of Engineering, Chulalongkorn University, Bangkok 10330, Thailand

**Keywords:** cell spheroid, embryoid body, microfluidics, microwells, perfusion, pyramidal tip angles

## Abstract

Three-dimensional (3D) tissue culture models provide in vivo-like conditions for studying cell physiology. This study aimed to examine the efficiency of pyramidal microwell geometries in microfluidic devices on spheroid formation, cell growth, viability, and differentiation in mouse embryonic stem cells (mESCs). The static culture using the hanging drop (HD) method served as a control. The microfluidic chips were fabricated to have varying pyramidal tip angles, including 66°, 90°, and 106°. From flow simulations, when the tip angle increased, streamline distortion decreased, resulting in more uniform flow and a lower velocity gradient near the spheroids. These findings demonstrate the significant influence of microwell geometry on fluid dynamics. The 90° microwells provide optimal conditions, including uniform flow and reduced shear stress, while maintaining the ability for waste removal, resulting in superior spheroid growth compared to the HD method and other microwell designs. From the experiments, by Day 3, spheroids in the 90° microwells reached approximately 400 µm in diameter which was significantly larger than those in the 66° microwells, 106° microwells, and HD cultures. Brachyury gene expression in the 90° microwells was four times higher than the HD method, indicating enhanced mesodermal differentiation essential for cardiac differentiation. Immunofluorescence staining confirmed cardiomyocyte differentiation. In conclusion, microwell geometry significantly influences 3D cell culture outcomes. The pyramidal microwells with a 90° tip angle proved most effective in promoting spheroid growth and cardiac differentiation of mESC differentiation, providing insights for optimizing microfluidic systems in tissue engineering and regenerative medicine.

## 1. Introduction

Three-dimensional (3D) tissue culture has become an important bridge between in vitro and in vivo models in various biological research fields [1,2]. Three-dimensional structures provide in vivo-like conditions that are beneficial for studying cell physiology and conducting high-throughput drug discovery and screening [3,4,5]. In 3D clusters like spheroids, cells communicate with each other and the extracellular matrix [6,7]. However, static culture systems, while simple and cost-effective, are limited in replicating key cell–environment interactions such as oxygen delivery, nutrient exchange, and waste removal [8,9]. To address these limitations, bioreactors with continuous fluid flow have been developed to improve 3D culture conditions. These bioreactors allow for precise control over fluid dynamics, gas exchange, and mechanical stimuli like shear force [10,11]. This approach has been successful in producing cardiomyocytes in both mouse and human models [12,13]. However, large-volume bioreactors can be costly, especially when significant amounts of chemicals or growth factors are required [14,15].

Microfluidic systems offer a more cost-effective alternative, using small volumes of fluid to control 3D tissue aggregation under precise conditions [16,17]. These systems have been applied to create “heart-on-chip” models for high-throughput drug testing [18]. Differentiation of mouse embryonic stem cells (mESCs) into cardiomyocytes has been a focal point of these studies [19]. Traditionally, the gold standard for cardiac differentiation is cell aggregation using the hanging drop (HD) method [20,21,22,23]. While effective, this method is labor-intensive and time-consuming [24].

To streamline cell aggregation, automated lab-on-chip technology using microfluidics has been developed. This technology controls small fluid volumes in channels, simulating physiological conditions on a microscale [25,26]. However, several factors influence the effectiveness of spheroid formation in microfluidic systems, including flow shear stress [27], cell type [28], and microwell shapes [29]. For efficient cell aggregation, the microwell platform integrated with microfluidic systems must ensure uniform spheroid size, reproducibility, and the ability to monitor the process [30,31].

The microwell shape significantly impacts cell culture outcomes. Pyramidal microwells have shown promise in reducing cell loss and guiding cells to the bottom, where spheroids are formed [32,33,34]. Inclined walls help direct cells into aggregation zones, minimizing the removal of unwanted cells during experiments [35]. Parameters such as the surface area-to-volume (SAV) ratio and tip angle affect how cells aggregate and how nutrients and oxygen are distributed. The pyramidal microwells with acute angles can create stronger flow disturbances, which may aid nutrient transfer but also increase mechanical stress on cells [36]. Designing effective microwell shapes requires balancing nutrient and oxygen transfer with controlled flow disturbances, such as recirculating flow velocity inside microwells [37]. Using arrays of pyramidal microwells increases surface area while decreasing SAV, creating better conditions for dynamic cell culture [38,39].

This study investigates how different pyramidal angles of microwells affect flow recirculation, cell spheroid formation, and viability in mESCs. The study focuses on flow characteristics, spheroid morphology, cell density, and viability during culture, aiming to optimize microwell design for improving 3D tissue engineering.

## 2. Materials and Methods

All chemicals were obtained from Sigma Aldrich (St. Louis, MO, USA) unless stated otherwise.

### 2.1. Fabrication and Design of Microfluidic Device

**Figure 1:**
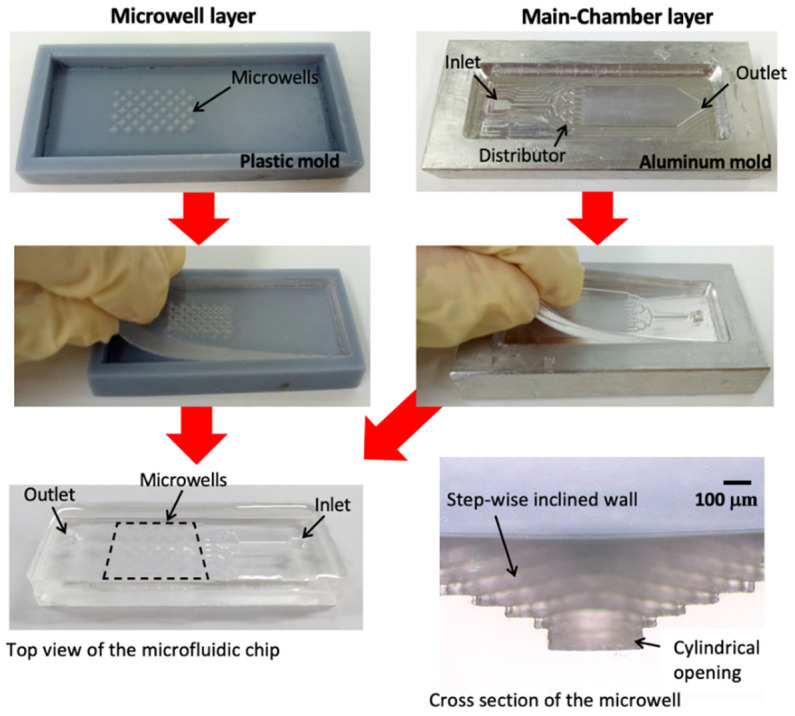
Fabrication of the PDMS microfluidic chip using 3D-printed plastic and aluminum molds. The fabricated device, measuring 22 mm in length and 12.5 mm in width, features an array of microwells with varying pyramidal tip angles (66°, 90°, and 106°) and 550 µm in depth.

**Figure 2:**
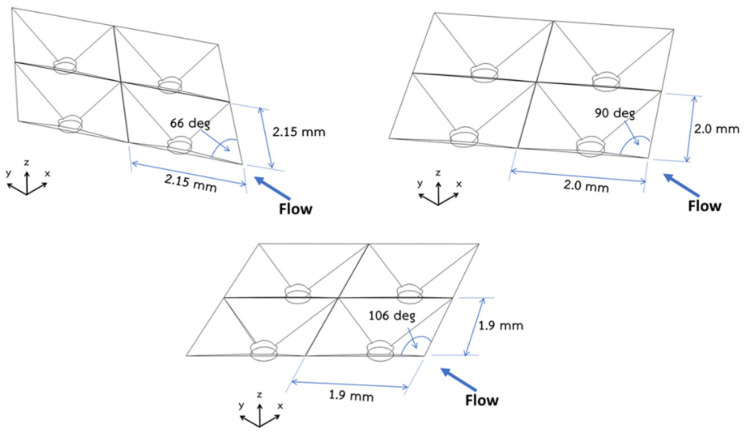
Schematic illustration of the pyramidal microwells with varying tip angles such as 66°, 90°, and 106°. The direction of the main flow is indicated by arrows.

The microfluidic chips were fabricated using a standard soft-lithography process consisting of two parts: the microwell layer and the main chamber layer. The microwell layer was created using a 3D-printed plastic mold, while the main chamber layer was formed with a machined aluminum mold. Poly-dimethylsiloxane (PDMS) mixed with a curing agent at a 10:1 ratio was carefully poured into the molds to avoid air bubble formation. After the PDMS solidified, the microwell layer, main chamber layer, and tube connections were bonded together using oxygen plasma bonding. Figure 1 shows the fabrication process of a PDMS microfluidic chip using 3D-printed plastic and aluminum molds. The microwells had varying pyramidal tip angles, including 66°, 90°, and 106°, as shown in Figure 2. The SAV ratio for all cases ranged between 6.67 and 6.70. The microwells with 66° and 90° angles have a foreplan area of 4.22 mm^2^ and 4.00 mm^2^, respectively, while the 106° microwell has a foreplan area of 3.69 mm^2^. This design enables the investigation of geometry effects on fluid dynamics and cell culture.

### 2.2. Flow Simulation and Fluid Shear Stress

A computational fluid dynamics (CFD) model was developed using COMSOL Multiphysics 5.3 software (COMSOL Inc., Burlington, MA, USA) to analyze velocity distribution, wall shear stress, and shear stress around the spheroids in pyramidal microwells. The steady-state Navier–Stokes and convection–diffusion equations were solved, assuming an incompressible, isothermal Newtonian fluid (fluid density = 998.2 kg/m^3^, viscosity = 0.001 Pa·s), with no-slip conditions at the walls. The microfluidic channel and four microwells were simulated at a flow rate of 10 μL/h (or approximately 1 µm/s inlet velocity). Cell spheroids with fixed diameters of 200 µm were placed inside the microwells, allowing for a detailed analysis of shear stress distribution. This setup was chosen to mimic realistic experimental conditions and approximate the magnitude of velocity and shear stress that would affect cell viability and spheroid function.

### 2.3. Culture of Mouse Embryonic Stem Cells

The mESCs from the 129S2/SvPas D3 line (ATCC, Washington DC, USA) were cultured on mitomycin C-treated mouse embryonic fibroblasts (MEFs), which served as a feeder layer to support stem cell maintenance. The mESCs were maintained in an ES cell culture medium consisting of high-glucose Dulbecco’s Modified Eagle Medium (DMEM) supplemented with 10% ES-grade fetal bovine serum (FBS) (Gibco, Waltham, MA, USA), 1000 IU/mL mouse leukemia inhibitory factor (LIF) (Millipore, Burlington, MA, USA), 0.1 mM β-mercaptoethanol (β-ME), 0.1 mM non-essential amino acids (NEAAs), and 1% antibiotic–antimycotic solution (Anti-Anti).

### 2.4. Spheroid Formation and Cardiac Differentiation

Two different approaches were employed for the spheroid formation of mESCs such as HD and microwell (cell-on-chip) methods. The mESCs were cultured to approximately 80% confluence and then trypsinized with 0.25% trypsin–EDTA to produce a single-cell suspension. The cells were counted using a hemocytometer and diluted to the appropriate concentration for differentiation. The differentiation medium consisted of an ES medium with 15% defined FBS (HyClone™, Omaha, NE, USA), excluding LIF. For the HD method, 20 µL drops containing 800 dissociated mESCs were placed on the lid of a bacterial Petri dish, with phosphate-buffered saline (PBS) in the base to prevent evaporation. Cell aggregation occurred via gravity, leading to the spontaneous formation of spheroids. The formation of spheroids was observed and monitored using a phase contrast microscope (Olympus CKX41, Tokyo, Japan).

**Figure 3:**
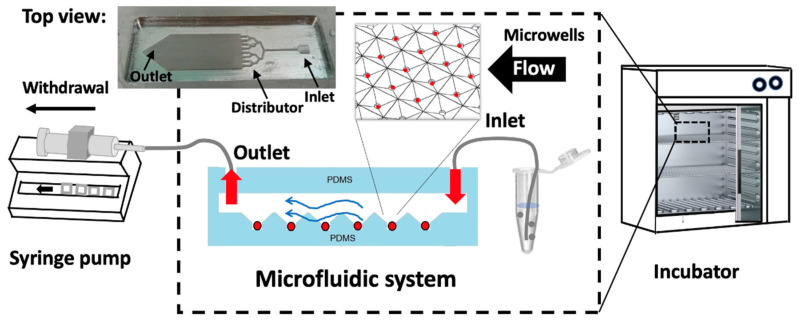
Schematic of the experimental setup for spheroid formation using the microfluidic system installed inside a CO_2_ incubator.

For cardiac differentiation using the microwells in microfluidic devices, pyramidal microwells were filled with a culture medium by injecting it slowly through the microchannel inlet using a micropipette. A cell suspension of 95 µL, containing 1 × 10^5^ mESCs, was introduced, resulting in an approximated cell density of 800 cells per well. The cells gradually settled into the microwells via gravity. The microfluidic system was then connected to a silicone tube and syringe pump operating in withdrawal mode, as shown in Figure 3, with a fresh culture medium supplied at a continuous flow rate of 10 μL/h for three days. A fresh culture medium was introduced through the inlet, and fluid was withdrawn through the outlet. All differentiation experiments were conducted in a humidified incubator at 37 °C with 5% CO_2_.

### 2.5. Spheroid Morphometry and Viability Analysis

To assess spheroid health and viability under varying flow conditions through morphometric analysis and live/dead cell viability assays. Spheroids were imaged using a brightfield microscope (Olympus CKX41, Tokyo, Japan) to capture their morphology. Spheroid diameters and circularity were measured using ImageJ software (ImageJ 1.x), ensuring precise quantification of their structural properties.

For the viability assessment, spheroids were cultured for three days and then harvested from the microwells. The spheroids were dissociated into single cells using trypsin–EDTA treatment, followed by staining with Calcein Acetoxymethyl (Calcein AM) and Ethidium Homodimer-1 (EthD-1) for live/dead analysis. Fluorescent microscopy (Olympus BX51, Tokyo, Japan) was used to visualize the cells, with viable cells showing green fluorescence in the cytoplasm due to the retention of Calcein AM, while dead cells exhibited red fluorescence in the nucleus due to EthD-1 staining of compromised membranes. Fluorescent images were captured and analyzed to quantify the viability of cells within each spheroid.

### 2.6. Proliferative Activity Evaluation

Cell proliferation was assessed using the 3-(4,5-Dimethylthiazol-2-yl)-2,5-Diphenyltetrazolium Bromide assay (MTT) assay. Harvested spheroids were transferred to a 96-well plate, with one spheroid per well. Each well was filled with 200 µL of MTT solution (0.5 mg/mL in PBS). The spheroids were incubated in the dark for 30 min at 37 °C and 5% CO_2_. After incubation, the MTT solution was removed, and the formazan crystals formed in the cells were dissolved with 200 µL of DMSO per well. Optical density (OD) was measured at 570 nm, with a reference wavelength of 650 nm, using a microplate reader. The resulting absorbance values were compared to a standard curve to indirectly quantify the cell number, serving as a measure of proliferative activity within each spheroid.

### 2.7. Gene Expression Assessment

The expression of the Brachyury gene in spheroids was quantified using a quantitative real-time polymerase chain reaction (qRT-PCR). Total RNA was extracted from EBs using the RNeasy Mini Kit (Qiagen, Venlo, The Netherlands), following the manufacturer’s instructions. The extracted RNA was reverse-transcribed into complementary DNA (cDNA) using SuperScript III Reverse Transcriptase (Invitrogen, Waltham, MA, USA). qRT-PCR was performed using GoTaq^®^ Green Master Mix (M7122, Madison, Promega, WI, USA) on a CFX96 Connect™ Real-Time PCR System (Bio-Rad, Hercules, CA, USA). The specific primers used for Brachyury were as follows: Forward 5′-TACACCTCTAATGTCCTCCCTTG-3′ and Reverse 5′-CCATACAGTTGACTTCCCAACAC-3′. The housekeeping gene *GAPDH* was used as an internal control, with the following primers: Forward 5′-AATGTGTCCGTCGTGGATCT-3′ and Reverse 5′-CCTGCTTCACCACCTTCTTG-3′. Relative gene expression was calculated using the ΔΔCt method to normalize Brachyury expression levels against GAPDH.

### 2.8. Assessment of Cardiac Differentiation Efficiency

To evaluate cardiac differentiation efficiency, the percentage of beating spheroids was determined. Spheroids harvested from both HD cultures and 90° microwells (the optimal condition identified in previous experiments) were cultured in a cardiac differentiation medium for fourteen days. The number of beating-cell colonies was counted. The differentiation medium consisted of high-glucose DMEM (Cat. No. SH30022.22, HyClone™, Omaha, NE, USA) supplemented with 15% defined fetal bovine serum (Cat. No. SH30070.03, HyClone™, Omaha, NE, USA), 1% antibiotic–antimycotic, 1% L-glutamine, 1% MEM non-essential amino acids (NEAAs, Cat. No. M7145, Sigma Aldrich, St. Louis, MO, USA), 10 ng/mL Bone Morphogenetic Protein 4 (Recombinant Human BMP-4, Cat. No. 4578, BioVision, Exton, PA, USA), 50 ng/mL Ascorbic Acid (Cat. No. A4544, Sigma Aldrich, St. Louis, MO, USA), and 25.9 mM beta-mercaptoethanol (Cat. No. 21985-023, Gibco, NY, USA).

### 2.9. Immunofluorescence Staining Test

Mouse cardiomyocytes were fixed in 4% (*v*/*v*) paraformaldehyde for 15 min at room temperature, followed by three washes with phosphate-buffered saline (PBS). Permeabilization was performed using 0.1% Triton-X 100 in PBS containing 2% bovine serum albumin (BSA) to block nonspecific binding sites. The cells were then incubated overnight at 4 °C with mouse anti-cardiac Troponin T (cTnT) primary antibodies (1:100; ab33589, Abcam, Cambridge, UK) to specifically label cardiomyocytes. After primary antibody incubation, the cells were washed and incubated with TRITC-conjugated secondary antibodies (1:100; T5393, Sigma Aldrich, St. Louis, MO, USA) for 1 h at 37 °C. The 4′,6-diamidino-2-phenylindole (DAPI) staining was applied for 15 min at room temperature to label the nuclei. The samples were then washed and imaged using a fluorescence microscope (Olympus BX51, Tokyo, Japan), capturing both cardiomyocyte-specific and nuclear staining.

### 2.10. Statistical Analysis

Statistical analysis was performed using SPSS version 29. A normality test was conducted with a 95% confidence level, followed by one-way analysis of variance (ANOVA) and post hoc Bonferroni tests, to determine significant differences between groups. Data are represented as mean ± standard deviation, with differences considered significant at *p* < 0.05.

## 3. Results

### 3.1. Pyramidal Microwells

The roughness of the microwell surfaces was measured using a Surface Roughness Tester (Mitutoyo SV-3000, Kanagawa, Japan). The measured roughness was 0.395 ± 0.078 µm. The inclined surfaces of the microwells exhibited a stepwise structure with an average width of 90.45 ± 4.56 µm and a height of 45.58 ± 1.56 µm. The diameters of the cylindrical openings at the center were 446.96 ± 4.03 µm, 449.43 ± 5.22 µm, and 453.15 ± 2.80 µm for the 66°, 90°, and 106° microwell configurations, respectively. The microwell arrays consisted of 45 wells for the 66° tip angle, 42 wells for the 90° tip angle, and 52 wells for the 106° tip angle. These geometric and structural variations were critical for analyzing the effects of microwell shape on fluid dynamics and cell behavior within the microfluidic system.

### 3.2. Velocity Distribution and Fluid Shear Stress

**Figure 4:**
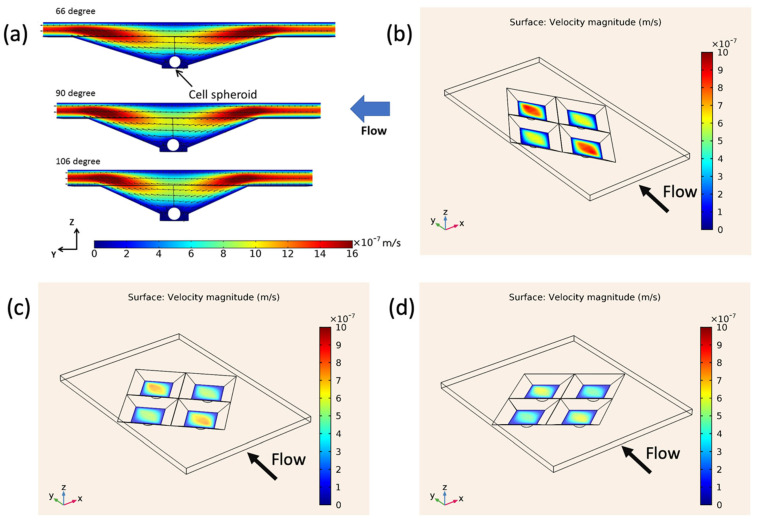
Velocity distribution in microwells with pyramidal tip angles of 66°, 90°, and 106°: (**a**) side view of velocity contour displaying flow dynamics around embedded cell spheroids and (**b**–**d**) top views of velocity contours for 66°, 90°, and 106° microwells at a flow rate of 10 µL/h.

Figure 4 illustrates the velocity distribution above cell spheroids in microwells with tip angles of 66°, 90°, and 106°. As the tip angle increased, streamline distortion decreased, resulting in more uniform flow and lower velocities near the spheroids. The 106° microwell exhibited the most uniform flow, indicating a stable environment that could reduce shear stress on cells. The velocity at the center of the 66° microwell was approximately double that of the 106° microwell, highlighting the concentrated flow in narrower tip angles. This similar flow pattern was observed at higher flow rates with velocity magnitudes increasing proportionally with higher inlet velocities. Near the spheroids, the velocity was around 1 × 10^−7^ m/s at a flow rate of 10 μL/h. These findings demonstrate the significant influence of microwell geometry on fluid dynamics, with wider angles probably promoting more favorable conditions for cell culture by lowering flow disturbance and minimizing fluid shear stress.

**Figure 5:**
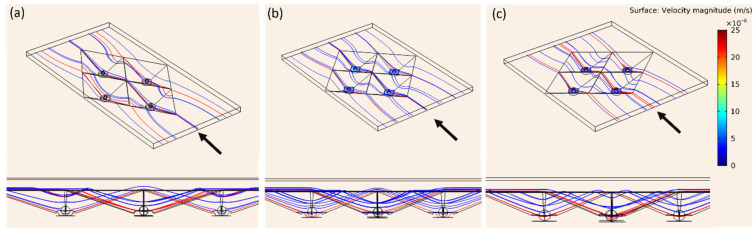
Distribution of streamlines in microwells with tip angles of (**a**) 66°, (**b**) 90°, and (**c**) 106°. The flow direction is indicated by the arrows.

The flow streamlines through each microwell are depicted in Figure 5. Red streamlines represent flow near the cell spheroids at the microwell bottom, while blue streamlines indicate flows that do not reach the bottom region. In all configurations, only the central flow passes directly above the spheroids. The 106° microwell shows more uniform flow near the spheroids, whereas narrower angles, like the 66° microwell, display more confined and distorted flow patterns. These findings emphasize the role of microwell geometry in shaping flow behavior, with wider tip angles facilitating straight and uniformly distributed flows, potentially reducing shear stress on the cells. On the other hand, the ability for waste removal and nutrient transfer might be weakened.

**Figure 6:**
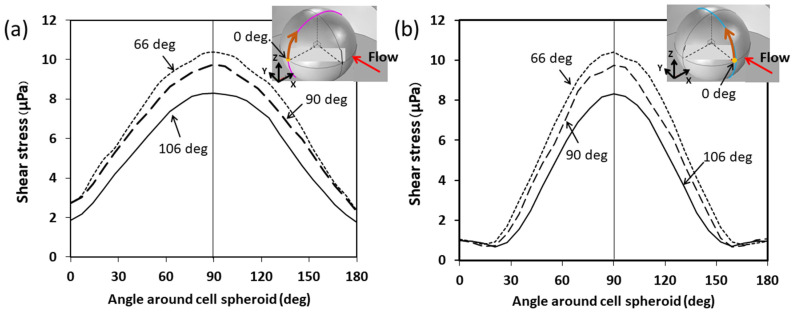
Distribution of shear stress on the surface of cell spheroid for microwells with tip angles of 66°, 90°, and 106°: (**a**) shear stress along the spanwise direction and (**b**) shear stress along the streamwise direction.

The distribution of shear stress on the surface of the cell spheroid is shown in Figure 6. This finding reveals a gradual increase in shear stress in both the streamwise and spanwise directions as the angle around the spheroid increases. The maximum shear stress occurs at the top of the spheroid (at 90°), then gradually decreases again. Narrower microwell angles (e.g., 66°) generate higher shear stress compared to wider angles (e.g., 106°), where the shear stress is more evenly distributed. These results show the influence of microwell geometry affecting the distribution of shear stress, which is crucial for maintaining the viability and integrity of cell spheroids.

### 3.3. Cell Growth and Morphological Progression of mESC-Derived Spheroids

After gently seeding cell suspensions, we supplied the medium at a constant flow rate to facilitate even deposition of cells into the microwells. After that, the cells were allowed to rest for 120 min to enable self-aggregation. Subsequently, we harvested the cells from the microwells using a micropipette and assessed the cell count using the MTT assay. The cell numbers were quantified based on a standard curve generated from the MTT assay. We have checked this procedure with other cells with imaging techniques as well before conducting this research, and it was found that the number of cells in each microwell is quite uniform. The variation is 5–10% of the mean value.

**Figure 7:**
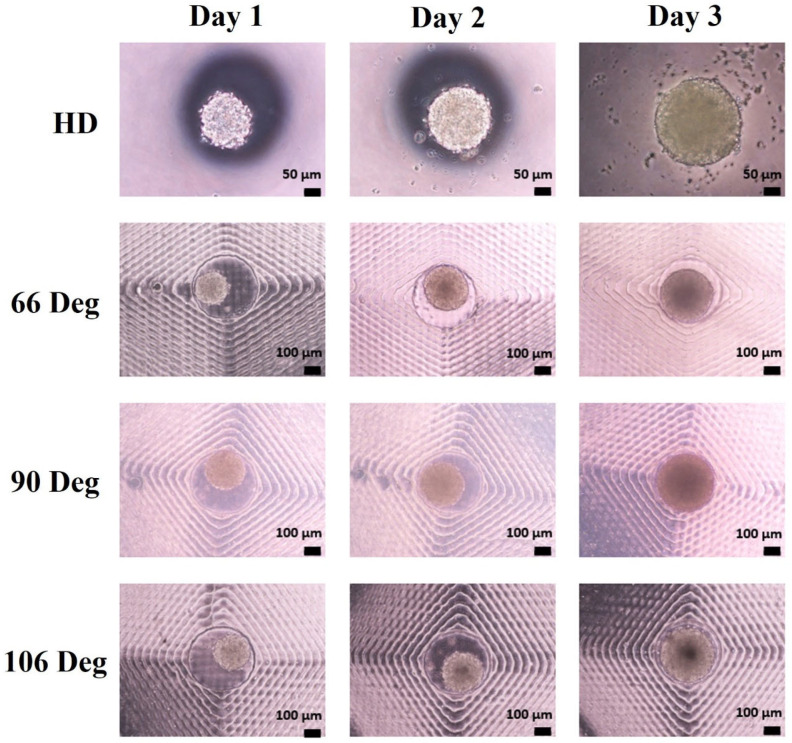
Morphological progression of mESC-derived spheroids cultured in pyramidal microwells with tip angles of 66°, 90°, and 106°, compared to static culture in HD. Images were captured on Day 1, Day 2, and Day 3. Scale bars represent 50 µm for the HD images and 100 µm for the microwell images.

**Figure 8:**
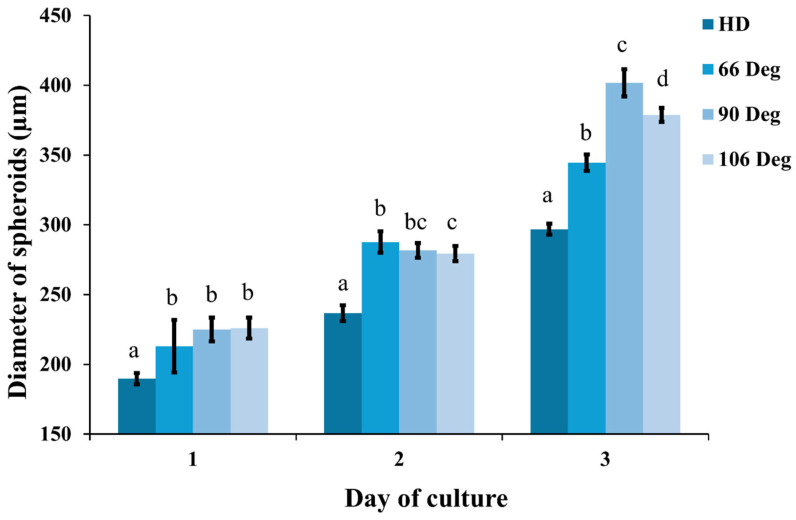
Spheroid size measured over three days across different culture methods (*n* = 10 per group). Statistical significance between groups is indicated by different letters (a–d), with *p* < 0.05.

In our experiments, the initial number of mESCs seeded on Day 0 in the 66°, 90°, and 106° microwells was 831 ± 89, 876 ± 103, and 876 ± 103 cells per well, respectively. Over the three-day period, all microwell configurations showed an increase in cell growth and viability, with the spheroids in the 90° microwells consistently exhibiting larger growth compared to the 66° and 106° microwells, as well as the static HD culture. As shown in Figure 7, the morphology of the mESC-derived spheroids varied with microwell geometry. On Day 1, spheroids across all microwell angles were relatively uniform in size. By Day 2, spheroids in the 90° microwells began to show more rapid expansion, but still being comparable to those in the 66° and 106° microwells. By Day 3, spheroids in the 90° microwells exhibited the largest growth, reaching diameters of around 400 µm, which was significantly larger than those formed in the other microwell configurations, as depicted in Figure 8.

It is quite common to observe a darkened center in cell aggregates, as the innermost cells become packed into a dense mass. This high cell density can limit oxygen and nutrient diffusion into the core of the aggregate. Additionally, as cells aggregate into a 3D structure, the center becomes more compact, increasing cell concentration. This denser region may scatter light differently, contributing to the darker appearance at the spheroid’s center. The darkened center of cell aggregates can influence cardiac differentiation, reflecting processes such as hypoxia, metabolic changes, and apoptosis, which are characteristic of the early stages of cardiac cell development.

These results suggest that the 90° microwells provide optimal conditions for spheroid formation. Additionally, nearly 100% cell viability was observed across all microwell configurations, indicating successful culture and growth of the spheroids. This trend reflects the correlation between the microwell angle and cell growth, with the 90° tip angle providing optimal conditions for spheroid expansion.

### 3.4. Proliferative Activity

**Figure 9:**
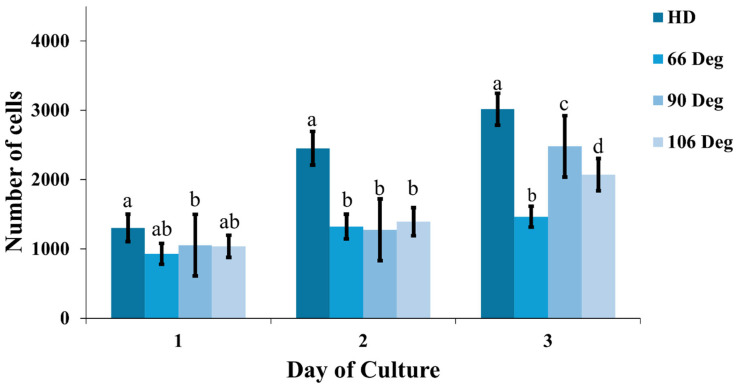
Cell proliferation across different culture methods over three days (*n* = 10 per group). The graph shows the number of cells on Days 1, 2, and 3 for HD cultures, and 66°, 90°, and 106° microwell configurations. Statistical significance between groups is indicated by different letters (a–d), with *p* < 0.05.

The MTT assay was used to quantify cell proliferation in each spheroid. On Day 1, all microwell configurations showed similar cell numbers, with no significant differences. By Day 2, all microwells did not show significant cell growth compared to the HD method, which had faster proliferation rates. However, by Day 3, cell numbers increased to 1500–2000 per well across all microwells, with the 90° configuration showing the most significant growth as shown in Figure 9. Despite these results, the overall growth rates for three days in all microwells were slower than those in the HD method.

Compactness was further estimated as the ratio of the number of cells to the cube of the spheroid diameter. On Day 2, compactness was measured at 0.186 ± 0.038 for the HD, 0.056 ± 0.011 for the 66° microwells, 0.058 ± 0.012 for the 90° microwells, and 0.048 ± 0.010 (×10^−3^ μm^−3^) for the 106° microwells, with an uncertainty of approximately 20% for all cases. These results indicate that conventional techniques as the HD method resulted in denser cell spheroids. Additionally, the spheroids in the 106° microwells were relatively loose, while the 66° and 90° microwells produced spheroids with comparable compactness. By Day 3, all spheroids continued growing, with compactness decreasing to 55–60% of Day 2 levels.

### 3.5. Gene Expression

**Figure 10:**
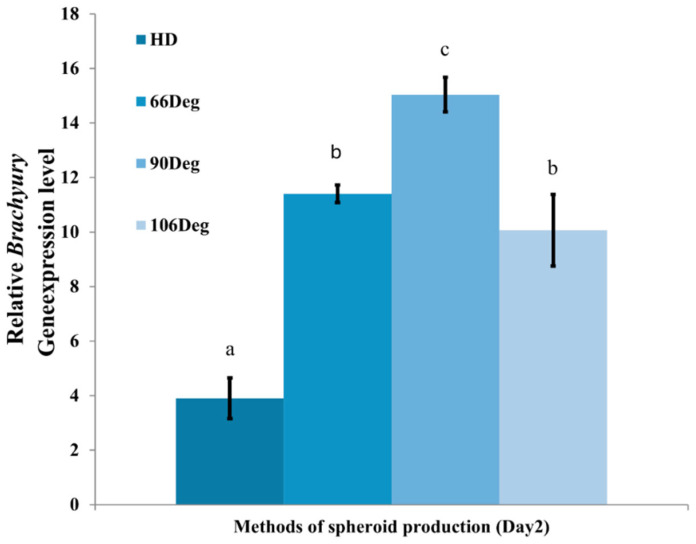
Comparison of Brachyury gene expression during differentiation on Day 2 across all spheroid formation methods (*n* = 3 per group). Statistical significance between groups is indicated by different letters (a–c), with *p* < 0.05.

On Day 2, Brachyury gene expression analysis revealed that cells cultured in the 90° microwells exhibited significantly higher expression levels compared to the other methods as shown in Figure 10. Specifically, the expression in the 90° microwells was approximately four times greater than that observed in the HD method, indicating a notable enhancement in mesodermal differentiation.

### 3.6. Immunofluorescence Staining

**Figure 11:**
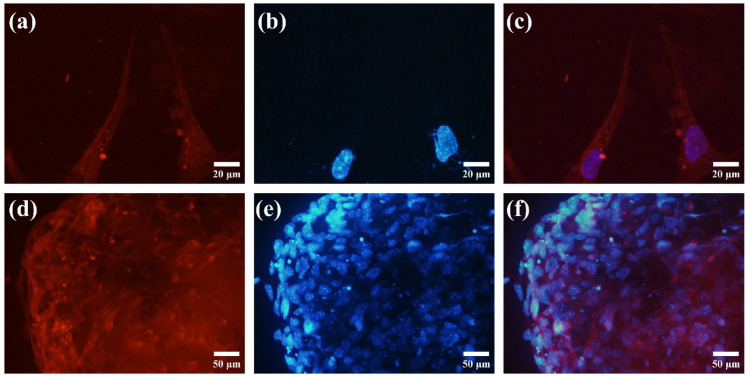
Immunofluorescence staining of mESC differentiation into cardiomyocytes: (**a**,**d**) cardiac Troponin T expression is indicated by bright red fluorescence, confirming the presence of cardiomyocytes; (**b**,**e**) DAPI staining highlights the nuclei as bright blue spots, providing a clear visualization of cell nuclei; and (**c**,**f**) merged images of Troponin T and DAPI staining. Figures (**a**–**c**) represent single cells, while figures (**d**–**f**) represent plated aggregate cells.

Immunofluorescence staining confirmed the differentiation of mESCs into cardiomyocytes. Cardiac Troponin T, a key marker of cardiomyocytes, was observed as bright red fluorescence in individual cells and larger clusters (Figure 11a,d), validating successful cardiac differentiation. DAPI staining (Figure 11b,e) highlighted cell nuclei as bright blue spots, revealing the organization and density of the culture. Merged images shown in Figure 11c,f confirmed the co-localization of Troponin T with cell nuclei, further supporting effective cardiomyocyte formation.

## 4. Discussion and Conclusions

This study highlights the significant impact of microwell tip angles on spheroid formation, cell viability, and differentiation of mESCs into cardiomyocytes. The acute angles, particularly in the 66° microwells, produced higher flow velocities in the microwell, which may have countering effects on spheroid formation. On one hand, higher flow velocities may enhance nutrient and waste transport, allowing cells to accumulate efficiently at the bottom of the microwells. On the other hand, the continuous strong flow may disrupt cell–cell interactions, hindering proper spheroid formation by interrupting cell communication. These results are consistent with previous findings that higher flow velocities can lead to hydrodynamic disturbances, affecting tissue morphogenesis in microfluidic systems [40,41,42].

Our findings showed that the 90° microwell configuration emerged as the optimal environment for promoting spheroid growth and differentiation. The balanced flow dynamics in these microwells provided adequate nutrient exchange without introducing excessive shear stress, leading to larger spheroids and enhancement of cell proliferation by Day 3. The continuous flow improved nutrient transfer and waste removal, promoting higher cell viability and consistent spheroid expansion [43]. While the 66° and 106° microwells also supported spheroid formation, the spheroids were either too dense or too loose, limiting optimal growth. The 66° microwells, in particular, had the highest shear stress, and high shear stress could disrupt the extracellular matrix (ECM) and suppress proliferation but promote cell apoptosis, resulting in slower growth rates and inhibition of cell aggregation [44,45].

The influence of microwell geometry on shear stress was evident, as narrower tip angles (66°) generated higher shear stresses near the cell spheroids, while the 106° microwells produced lower and more uniform shear stress distributions around the cell spheroids. Although shear stress value is a critical factor in cellular differentiation and tissue formation [46], non-uniform shear stress in the narrower microwells likely contributed to less homogeneity in spheroid structure and function [47]. Our results suggested that relatively uniform shear stress, as seen in the 90° microwells, plays a key role in facilitating optimal nutrient distribution and promoting uniform cell growth and differentiation.

Interestingly, spheroid compactness was influenced by both the microwell geometry and the dynamic culture conditions. Looser spheroids were observed in all microwells, likely due to hydrodynamic forces affecting cell–cell interactions and ECM remodeling. This observation is consistent with several studies that suggest dynamic cultures typically produce looser aggregates, whereas static cultures, with slower diffusion rates, tend to form more compact spheroids [48]. By Day 3, compactness decreased across all microwell configurations, indicating that as the spheroids expanded, the cells became more distributed within the aggregate.

A key finding in our study was the significant elevation of Brachyury gene expression, a marker for mesodermal differentiation, in the 90° microwells. The expression levels in these microwells were approximately four times higher than in the HD method. This enhanced gene expression in the 90° microwells can be attributed to the favorable flow dynamics, which facilitated both nutrient transfer and mechanical stimulation, two critical factors known to drive differentiation to special tissues. Our results align with previous studies that highlight the importance of dynamic environments for enhancing cell differentiation due to improved nutrient exchange and mechanical cues [49].

It should be noted that Brachyury is an early mesodermal marker and not specific to cardiac cells. That is why we examined gene expression as early as Day 2 after spheroid plating. However, the formation of the mesoderm is crucial for cardiac differentiation. Therefore, a higher expression of mesodermal lineage markers is often correlated with the differentiation potential of cardiac cells.

Although spheroid formation and differentiation were successful, cardiomyocyte differentiation efficiency in the microwells with the 90° tip angle was lower than the HD method. This may be attributed to the looser structure of the spheroids, which could lead to cell damage during harvesting [50]. Additionally, differences in differentiation efficiency could result from changes in spheroid size, exposing cells to varied microenvironments [51,52]. However, cardiac Troponin T staining confirmed cardiomyocyte differentiation in all microwell configurations, highlighting the microfluidic system’s capability to produce functional cardiomyocytes.

The study also points to the importance of microwell design in optimizing differentiation outcomes. The microwells with a 90° tip angle, due to their balanced shear stress and nutrient transfer, provided the most favorable conditions for mESC differentiation into mesodermal and cardiomyocyte lineages. In contrast, the 66° microwells, while effective at promoting initial cell accumulation, likely introduced excessive shear stress that interfered with ECM formation and cell aggregation. Meanwhile, the 106° microwells, although generating more uniform shear stress, did not promote robust spheroid growth or differentiation, likely due to less efficient nutrient exchange and waste removal.

In conclusion, this study underscores the critical role of microwell geometry in influencing cell behavior in microfluidic systems. The 90° microwell configuration proved to be the most effective for promoting spheroid growth, maintaining cell viability, and enhancing mesodermal differentiation, as evidenced by elevated Brachyury expression and successful cardiomyocyte formation. These findings provide valuable insights into the design of microfluidic systems for stem cell research and tissue engineering, particularly for applications requiring controlled differentiation and 3D cell culture. Further optimization of spheroid harvesting and compactness could improve differentiation efficiency, making the microwell system even more versatile for regenerative medicine and cardiac tissue engineering.

## Data Availability

The data that support the findings of this study are available from the corresponding author upon reasonable request.
